# Alcohol Dressings

**Published:** 1912-01

**Authors:** 


Alcohol Dressings.—A writer in *Therapie der Gegenwart* recommends for phlegmon, furunculosis, erysipelas, gout, arthritis, neuralgia, herpes zoster, etc., the use of dressings with gauze dipped in 70 to 90 per cent alcohol, the gauze being folded eight or ten times and covered with waterproof tissue.—*Medical Summary.*

